# Emotional and economic intimate partner violence as key drivers of depression and suicidal ideation: A cross-sectional study among young women in informal settlements in South Africa

**DOI:** 10.1371/journal.pone.0194885

**Published:** 2018-04-16

**Authors:** Andrew Gibbs, Kristin Dunkle, Rachel Jewkes

**Affiliations:** 1 Gender and Health Research Unit, South African Medical Research Council, Pretoria, South Africa; 2 Health Economics and HIV/AIDS Research Division (HEARD), University of KwaZulu-Natal, Durban, South Africa; 3 School of Public Health, University of Witwatersrand, Johannesburg, South Africa; Stellenbosch University, SOUTH AFRICA

## Abstract

Little research has assessed the impact of emotional intimate partner violence (IPV) and economic IPV on women’s mental health. Using cross-sectional data from the Stepping Stones and Creating Futures intervention trial baseline, in eThekwini Municipality, South Africa we assess three questions. First, whether emotional IPV and economic IPV make independent contributions to mental health outcomes; second what matters, severity, variety, or absolute experience? and third, are some items more important in driving mental health impacts than others? We assess associations between past 12-month emotional IPV, past 12-month economic IPV, and past week depressive symptoms and past four-week suicidal ideation. We describe the prevalence of each mental health outcome by individual items, including never/ever and frequency, and combined emotional IPV, and economic IPV, reporting depression scores and percentage of suicidal ideation and 95% confidence intervals (CI). Second, we created four-level categorical variables for combinations of emotional, economic, sexual and physical IPV, and present its frequency, and the mean/% and 95% CI for depression symptomatology and suicidal ideation. 680 women (aged 18–30) were enrolled. High levels of past year emotional IPV, economic IPV were reported. 45.3% reported clinically relevant symptoms of depression, and 30.0% past four-week suicidal ideation. All measures of emotional IPV and economic IPV showed a consistent positive correlation with CESD scores, and suicidal ideation. For all four-level categorical constructs the highest depression scores, and prevalence of suicidal ideation, were for combinations of emotional IPV or economic IPV with physical and/or sexual IPV. For depression in 17/18 combinations this was significantly different compared to women reporting no IPV. For suicidal ideation this was significant in 6/18 combinations all related to economic IPV. Emotional IPV and economic IPV have independent associations with women’s mental health, beyond physical IPV and sexual IPV, and also have distinct patterns between each other.

**Trial registration:**

NCT03022370. Registered 13 January 2017, retrospectively registered.

## Introduction

A decrease in emotional intimate partner violence (IPV) is now an indicator of progress for the Sustainable Development Goal 5.2. This indicator is one of 230 indicators assessing progress on the 17 global goals established by the UN to assess development progress by 2030 [1]. Yet comparatively little has been written on emotional IPV, and no consensus exists on the best practices for measuring emotional IPV, or understanding its impact on health outcomes. Overwhelmingly, research to date on IPV has focused on physical and/or sexual IPV [2]. While emotional–as well as economic–IPV are recognized as important components of women’s experiences of violence by survivors and advocates, they are less often included in research. Indeed, the Global Burden of Disease study in 2010 excluded emotional IPV and economic IPV as potential disease burdens because of the lack of research on these constructs and their connections to health impacts [3, 4]. The lack of research on emotional IPV and economic IPV is seen in other reviews. In 2012, a review of associations between IPV and suicide found that only 2 of 37 identified studies considered the differential impacts of emotional IPV on suicide [5]. Likewise, two recent reviews on associations between IPV and depression [6] and HIV [7], highlighted how few studies have examined the impact of emotional IPV, and they found no studies addressing economic IPV.

The lack of focus on emotional and economic IPV in research to date emerges for a number of reasons. First, there is a lack of consensus on best practices for measuring these constructs, which can manifest in myriad and culturally diverse ways [e.g. 2, 8], in contrast to physical and sexual IPV which can be more readily operationalized in questionnaires with standardized measures describing concrete behaviorally specific acts of assault [9, 10]. There also lingers a sense, despite first-hand account from survivors of IPV, that emotional and economic IPV are less severe than physical and sexual IPV, and have less impact on health.

Finally, there is also a lack of conceptual clarity about what constitutes emotional IPV and economic IPV. For emotional IPV, this is apparent in the multitude of terms for the same or similar phenomena, including psychological violence, or abuse. For clarity and a working definition in this paper, emotional IPV includes verbal abuse and humiliation, and threats of violence or other acts to scare a woman [2]. While economic IPV, constitutes men stopping women leading economic productive lives directly through either stopping them working, taking earnings, or forcing them from their houses. Through generating fear, isolating women, and removing autonomy from women’s lives, emotional IPV, and economic IPV, have the potential to impact on women’s mental health [11].

There is emerging evidence that the view that emotional and economic IPV have less health impact than physical or sexual IPV may be wrong. A review of research on IPV and suicide found two studies showing increased suicidal ideation and suicide attempts following emotional IPV, specifically Bangladeshi women were 2–3 times more likely to report suicidal ideation if they had experienced emotional IPV [5]. In a longitudinal study, Ludermir et al [12] found that the risk of post-natal depression (PND) following emotional IPV during pregnancy increased by aOR2.09, independently of physical and sexual IPV. A nationally representative study of women in Germany found that psychological IPV was significantly associated with past year psychosomatic conditions, such as numbness, shaking and nervous twitching, amongst younger (16–49) and older (50–65) women, and psychological problems, including depressive symptoms, amongst younger and older women [13]. In South Africa, among women living with HIV, emotional IPV was independently associated with immune system decline as measured through CD4 and CD8 cell counts[14]. Similarly, a longitudinal cohort analysis of young women in rural South Africa showed that depression was associated with emotional IPV, even after adjusting for the co-occurrence of physical and/or sexual IPV [15]. While these studies show consistent associations between emotional IPV and health outcomes, findings are tempered by the different ways in which emotional IPV is operationalized and analysed in each study.

There are even fewer studies exploring the health impacts of economic IPV, but those that do show that economic IPV also negatively impacts mental health. In Palestine, a nationally representative cross-sectional study showed women who experienced economic IPV were more likely to report depression and anxiety [11]. The nationally representative German study referenced above also showed older (50–65) women reporting economic IPV had increased psychosomatic symptoms, and younger (16–49) and older (50–65) reporting economic IPV had increased severe psychological problems [13].

While research on health impacts remains limited, it is clear that both economic and emotional IPV are widespread. A systematic review of emotional IPV in pregnancy in Africa estimated that 24.8% to 49% of pregnant African women experienced emotional IPV [16]. In the multinational WHO multi-country study, 20% to 75% of women in population-based surveys across 10 countries reported emotional IPV in the past year [9]. Data on the prevalence of economic IPV is more sparse. The majority of research on economic IPV comes from the global north, and often from studies of survivors living in shelters [17, 18]. From the global south, a nationally representative study in the Philippines suggested economic IPV ranged from 6.7% to 1.5% [19], while an estimate from Palestine found 44% of married women reported economic IPV [11]. The wide variation in estimates of prevalence of both emotional IPV and economic IPV is linked to different operationalization of constructs, and also likely how such forms of IPV occur in different populations.

There are a number of questions about how best to define and measure emotional and economic IPV, and no clear standard has yet emerged. The three major international surveys that have measured these constructs show slight, but important, variations. The WHO Multi-Country Study on Domestic Violence (WHO MCS), and the UN Multi-Country Study on Men and Violence in Asia and the Pacific (UNMCS) and the current Domestic Violence Module (Jan 2017) of the Demographic Health Survey (DHS) vary in the number of items used, and the focus and wording of questions (S1 Table).

Just as there is no consensus on the measurement of emotional and economic IPV, there is likewise no consensus on coding and analysis of responses to questionnaire items; this creates further complexities in understanding the prevalence and unique health impacts of these constructs. Some studies treat emotional and economic IPV as separate constructs, while others treat it as one construct [19–21]. Furthermore, it is not clear which dimensions of emotional IPV and economic IPV are most relevant for health outcomes: type of IPV as measured by questionnaire items or broad categories (i.e. emotional vs economic); timing of IPV (i.e. lifetime, past year, or another dimension); or frequency of IPV and, if so, over what time-period. Whilst the debates about measurement, coding, and data analysis continue unresolved, the concepts of emotional IPV and economic IPV are often pushed to the margins, undermining a comprehensive understanding of IPV. This situation is not tenable now emotional that IPV has been identified as an SDG indicator.

In this paper, we argue that through looking at the health impact of emotional IPV, and economic IPV, juxtaposed with the impact of physical and sexual IPV, we can begin to answer many of the central questions in the measurement of emotional and economic IPV. Building this understanding is important for understanding the relative health impact of different forms of IPV, developing and evaluating IPV prevention interventions, and understanding what is most useful to track for monitoring progress towards the SDG. We seek to answer three central questions. First, do economic and emotional IPV, make important, independent contributions to self-reported depressive symptomology and suicidal ideation? Second, if they are important, what matters in their measurement? Is it the number of types, number of episodes, or the overall severity of the IPV? Finally, are some items more important in driving health impacts than other items?

## Materials and methods

Between September 2015 and September 2016, 680 young adult women were recruited to participate in the Stepping Stones and Creating Futures cluster randomized control trial in urban informal settlements surrounding Durban, South Africa. 34 clusters were identified and community leaders approached for inclusion. In each cluster between 19 and 21 women were enrolled into the trial [22]. These areas are generally very impoverished with high levels of violence and residents experience many consequences of poverty and chronic stress, including poor mental health. The sample size was determined for the main trial outcome of IPV [22], as such, we did not do a specific sample size calculation for this analysis, which uses descriptive statistics, but given our estimates of IPV for the power calculation and inclusion of additional clusters and participants, to account for potential loss-to-follow-up, the sample size for this simple descriptive analysis is adequate.

Participants were aged between 18 and 30 and out of school. Further eligibility criteria included residence in the informal settlement and ability to understand the informed consent process. Participants were not blinded to study arm, those enrolling in the intervention arm received R100 (~US$7) and those in the control arm received R300 (~US$21) for completion of questionnaires. The structured questionnaires were self-completed on cellphones, with built in logic checks and skip patterns. Questionnaires were available in Zulu, Xhosa and English. Research staff matched to the participants by age and gender were available to clarify meaning or assist if literacy or technology posed an issue.

Participants provided informed written consent to participate. Ethical approval for the study was received from the University of KwaZulu-Natal, and the South African Medical Research Council. Further details on ethical procedures and research methods are described elsewhere [22].

### Measures

The content of the questionnaires was almost entirely based on surveys previously used and validated for use in South Africa. Our two key outcomes for this analysis were past week depressive symptomology and past month suicidal ideation.

### Depression and suicidal ideation

We assessed depressive symptoms using the 20-item The Centre for Epidemiologic Studies Depression Scale (CES-D) scale [23], which has been widely used in South Africa [24, 25]. Items ask about a variety of symptoms of depression in the past week, with responses rarely or none of the time, some or little of the time, moderate amount of time, or most or all the time. Women’s scores ranged from 0–60 (alpha = 0.88). A cut of ≥21 indicated probable depression [24], but for descriptive analysis a summative score was derived.

To measure past 4 week suicidal ideation, we asked a single item asked whether “the thought of ending your life been in your mind?” This approach has been used widely to assess suicidal ideation [26]. A binary response of yes/no was provided.

### Intimate partner violence

We assessed women’s experiences of four types of IPV in the past year: physical, sexual, emotional and economic. Questions on physical IPV were taken from the widely used South African adaption of WHO’s MCS survey [27]. Past year physical IPV was assessed through five behaviorally-specific items that included shoving, slapping, hitting, kicking, and being threatened or attacked with a gun or other weapon. Assessment of past -year sexual IPV was drawn from the WHO MCS [9] and covered physically forced and coerced sexual experiences from a current or previous partner in the past year. These items were initially developed in South Africa and are well adapted to this context [27]. Response choices for all items were never, once, few, many.

Emotional IPV in the past year was assessed using the five items from the WHO MCS survey [12] and adapted in previous studies for use in South African populations [27]. Two items asked about whether women had been insulted or humiliated by a partner in the past year, while three items sought to assess para-physical violence, such as threats to hurt the woman, scaring her, or hurting others of importance. Responses for each item were never, once, few, many.

Economic IPV was assessed using the four items from the UNMCS survey for women [21], which were in turn adapted from earlier work in South Africa [28]. The items covered actions by a current or former partner including preventing the woman from earning money, taking her money, throwing her out of the home, or spending money on alcohol, tobacco or himself when it was needed for the household. Response options were never, once, few, many.

### Analysis

Data were uploaded and compiled into a dataset for analysis in Stata IC 14.1. Because of self-completion on cellphones, and inbuilt logic checks, there was very little (<1%) data missing in the sample and no corrections were undertaken for this. Descriptive analyses took into account the clustered nature of the sample, and the 95% confidence intervals (CIs) were calculated using Taylor linearization. Taylor linearization corrects standard errors for the clustered nature of the sample, and provides wider CIs [29]. For each individual type of IPV (emotional, economic, physical, sexual) and for combined IPV exposures (emotional and/or economic, physical and/or sexual), we created a three-level categorical variable to indicate past year experience of IPV: never, once, more than once.

To describe associations between the emotional and economic IPV and our chosen mental health outcomes, we first described the prevalence of individual items of emotional IPV and economic IPV. For each item of the emotional and economic IPV scales we report the prevalence of, i) never/ever in the past 12 months, ii) two or more times in the past 12 months versus none or 1, and iii) the frequency of experience (never, once, few, many). We also report the prevalence of none versus any emotional or economic IPV and two or more versus none or one report of emotional or economic IPV. For each type of IPV described we present the mean and 95% CI for depression scores and the percentage and 95% CI for suicidal ideation.

To assess the associations between the four types of IPV exposures and depression/suicidal ideation, we first created a series of four-level categorical variable for a variety of combinations of emotional, economic, sexual and physical IPV and present variation in mental health outcome. For instance, we created a four-level variable for no exposure to violence, one or more emotional IPV exposures only, any emotional IPV and any physical IPV exposure, and physical IPV exposure only. This enabled the creation of ‘clean’ reference categories for each type of exposure, where typically there is overlap between types of violence that is not accounted for in analyses. We developed different permutations of this violence variable with physical IPV, sexual IPV, economic IPV, combined economic and emotional IPV and then examined if the effects were different if we considered never/ever exposures and higher versus lower frequency of exposure (two or more exposures). For each violence variable we present its frequency and the mean/% and 95% CI for depression symptomatology and suicidal ideation.

## Results and discussion

A total of 680 women were interviewed (Table 1). Women were aged between 18 and 30 with a mean age of 23.7 years. Only a third had completed high school (30.3%), while the majority (61.6%) had some secondary education. Most women were in a relationship, though the majority (64.9%) were not living with their partner. Just under a fifth (18.5%) reported they did not currently have a partner. One quarter (25.6%) had worked in the past three months, and earnings were similarly low.

**Table 1:** Descriptive statistics of sample demographics, IPV experiences and health outcomes.

Demographics	n	%/mean(CI95%)	
Age	680	23.7(23.4–23.9)	
Education			
Primary	55	8.1(6.3–10.4)	
Secondary	419	61.62(58.0–65.2)	
Completed high school	206	30.3(27.0–33.8)	
Relationship status			
Living with partner	113	16.6(14.2–19.5)	
Partner, but living separately	441	64.9(61.2–68.3)	
No partner	126	18.5(15.8–21.6)	
Work status			
Work in past 3 months (yes)	174	25.6(22.5–29.0)	
Earnings past month (Rand)	680	169(135–204)	
**Violence experiences**			
Physical IPV past 12m			
No experience	275	40.4(36.8–44.2)	
Once	75	11.0(8.9–13.6)	
Two or more	330	48.5(44.8–52.3)	
Sexual IPV past 12m			
No experience	480	70.6(67.0–73.9)	
Once	56	8.2(6.4–10.6)	
Two or more	144	21.2(18.2–24.5)	
Physical and/or sexual IPV past 12m			
No experience	237	34.9(31.3–38.5)	
Once	71	10.4(8.4–13.0)	
Two or more	372	54.7(50.9–58.4)	
Emotional IPV past 12m			
No experience	149	21.9(19.0–25.1)	
Once	79	11.6(9.4–14.3)	
Two or more	452	66.5(62.8–69.9)	
Economic IPV past 12m			
No experience	325	47.8(44.1–51.5)	
Once	58	8.5(6.6–10.9)	
Two or more	297	43.7(40.0–47.4)	
Emotional and/or economic IPV past 12m			
No experience	101	14.9(12.4–17.7)	
Once	60	8.8(6.9–11.2)	
Two or more	519	76.3(73.0–79.4)	
**Health outcomes**			
Depression			Range
Mean (95%CI)		21.2(20.4–22.0)	0–60
No (< = 20)	372	54.7(51.0–58.4)	
Yes (> = 21)	308	45.3(41.6–49.0)	
Past 4 week suicidal ideation			
No	476	70.0(66.4–73.4)	
Yes	204	30.0(26.6–33.6)	

All forms of IPV measured were highly prevalent (Table 1). Almost half of the women (48.5%) had experienced two or more instances of physical IPV in the past year, while a fifth had two or more experiences of sexual IPV in the past year. There were high overlaps between physical and/or sexual IPV with just over half (54.7%) reporting two or more experiences in the past year.

Two-thirds (66.5%) of the women reported two or more instances of emotional IPV in in the past year, while just under half (43.7%) reported two or more experiences of economic IPV in the past year. Three-quarters (76.3%) reported two or more experiences of emotional and/or economic IPV in the past year.

The mean CES-D score of women in the sample was 21.2. Almost half the sample (45.3%) reported potentially clinically relevant depression (a score of ≥ 21 [24]), while nearly one-third (30.0%) reported suicidal ideation in the last four weeks.

Tables 2 and 3 shows how various measures of emotional IPV and economic IPV–individual items, separate constructs (economic, emotional), and then a single combined construct–are associated with depressive symptoms or suicidal ideation. The most frequently reported form of emotional IPV (Table 2) in the past 12 months was being insulted or made to feel bad about oneself (64.7%), while hurting people she cared about as a way of hurting her, or damaging things of importance to her was least frequently reported (20.2%), but not uncommon. Being belittled or humiliated in front of others, threatened with violence, or scared/intimidated were reported by between 37.9–40.7% of women. In total, 78.1% of the women reported any emotional IPV in the past year, with 66.5% of the overall sample reporting two or more incidents, while 55.3% responded affirmatively to 2 or more of the 5 emotional IPV items.

**Table 2:** Descriptive associations between forms of emotional IPV and depressive symptoms and suicidal ideation.

		In the past 12 months how many times has a current or previous husband or boyfriend insulted you or made you feel bad about yourself?		In the past 12 months how many times has a current or previous husband or boyfriend belittled or humiliated you in front of other people?		In the past 12 months how many times has a current or previous husband or boyfriend done things to scare or intimidate you on purpose for example, by the way he looked at you, by yelling or smashing things?		In the past 12 months how many times has a current or previous husband or boyfriend threatened to hurt you?		In the past 12 months how many times has a current or previous husband or boyfriend hurt people you care about as a way of hurting you, or damaged things of importance to you?		Any emotional IPV in past 12m		2 or more experiences of emotional IPV in past 12m		Range of different forms of emotional IPV (None or one versus two or more)	
		Exposure prevalence % (n)	Symptom mean /%	Exposure prevalence % (n)	Symptom mean /%	Exposure prevalence %(n)	Symptom mean /%	Exposure prevalence %(n)	Symptom mean /%	Exposure prevalence %(n)	Symptom mean /%	Exposure prevalence %(n)	Symptom mean /%	Exposure prevalence %(n)	Symptom mean /%	Exposure prevalence %(n)	Symptom mean /%
Depression	Past 12m never	35.3(240)	18.63(17.4–19.9)	61.2(416)	19.6(18.6–20.5)	59.3(403)	19.0(18.0–20.0)	62.1(422)	19.0(18.1–20.0)	80.0(544)	20.2(19.3–21.1)	21.9(149)	17.9(16.3–19.5)			44.71(304)	18.1(17.0–19.2)
	Past 12m once or more	64.7 (440)	22.54(21.5–23.6)*	38.8(264)	23.7(22.3–25.1)*	40.7(277)	24.4(23.0–25.7)*	37.9(258)	24.7(23.3–26.1)*	20.0(136)	25.0(23.1–26.8)*	78.1(531)	22.1(21.2–23.0)*			55.29(376)	23.6(22.5–24.8)*
	Past 12m never or once	53.7(365)	18.8(17.8–19.7)	75.6(514)	19.7(18.8–20.6)	73.7(501)	19.5(18.6–20.3)	77.8(529)	19.6(18.7–20.4)	89.4(608)	20.4(19.6–21.3)			33.5(228)	18.0(16.8–19.3)		
	Past 12m twice or more	46.3(315)	24.0(22.7–25.2)*	24.4(166)	25.7(23.9–27.5)*	26.3(179)	25.9(24.2–27.7)*	22.2(151)	26.8(25.0–28.7)*	10.6(72)	27.5(24.8–30.2)*			66.5(452)	22.8(21.7–23.8)*		
	Frequency—never	35.3(240)	18.6(17.4–19.9)	61.2(416)	19.6(18.6–20.5)	59.3(403)	19.0(18.0–19.9)	62.1(422)	19.0(18.1–20.0)	80.0(544)	20.2(19.3–21.1)						
	Frequency—once	18.4(125)	19.0(17.3–20.7)	14.4(98)	20.3(18.4–22.2)	14.4(98)	21.6(19.5–23.6)	15.7(107)	21.6(19.6–23.7)	9.4(64)	22.1(19.7–24.5)						
	Frequency—few	23.1(157)	21.6(20.0–23.3)*	10.4(71)	24.1(21.5–26.7)*	14.196)	24.0(21.8–26.1)*	11.0(75)	23.8(21.7–26.0)*	5.9(40)	26.2(22.9–29.6)*						
	Frequency—many	23.2(158)	26.3(24.5–28.1)*	14.0(95)	26.9(24.4–29.4)*	12.2(83)	28.2(25.4–30.9)*	11.2(76)	29.8(27.0–32.6)*	4.7(32)	29.1(24.8–33.3)*						
Suicidal ideation	Past 12m never	35.3(240)	26.7(21.5–32.6)	61.2(416)	28.1(24.0–32.7)	59.3(403)	27.8(23.6–32.4)	62.1(422)	27.0(23.0–31.5)	80.0(544)	27.8(24.1–31.7)	21.9(149)	24.8(18.6–32.4)			44.71(304)	27.0(22.3–32.3)
	Past 12m once or more	64.7 (440)	31.8(27.6–36.4)	38.8(264)	33.0(27.6–38.9)	40.7(277)	33.2(27.9–39.0)	37.9(258)	34.9(29.3–40.9)	20.0(136)	39.0(31.1–47.5)	78.1(531)	31.5(27.6–35.6)			55.29(376)	32.5(27.9–37.4)
	Past 12m never or once	53.7(365)	27.4(23.0–32.3)	75.6(514)	28.4(24.6–32.5)	73.7(501)	30.0(26.1–34.1)	77.8(529)	28.5(24.8–32.6)	89.4(608)	28.3(24.8–32.0)			33.5(228)	27.6(22.2–33.8)		
	Past 12m twice or more	46.3(315)	33.0(28.1–38.4)	24.4(166)	34.9(28.1–42.5)	26.3(179)	30.2(23.9–37.3)	22.2(151)	35.1(27.9–43.0)	10.6(72)	44.4(33.4–56.1)*			66.5(452)	31.2(27.1–35.6)		
	Frequency—never	35.3(240)	26.7(21.5–32.6)	61.2(416)	28.1(24.0–32.7)	59.3(403)	27.8(23.6–32.4)	62.1(422)	27.0(23.0–31.5)	80.0(544)	27.8(24.1–31.7)						
	Frequency—once	18.4(125)	28.8(21.6–37.3)	14.4(98)	29.6(21.4–39.4)	14.4(98)	38.9(29.6–48.8)	15.7(107)	34.6(26.1–44.1)	9.4(64)	32.8(22.4–45.3)						
	Frequency—few	23.1(157)	28.7(22.1–36.3)	10.4(71)	29.6(20.1–41.2)	14.196)	25.0(17.4–34.6)	11.0(75)	30.7(21.3–42.0)	5.9(40)	47.5(32.7–62.7)*						
	Frequency—many	23.2(158)	37.3(30.2–5.1)	14.0(95)	39.0(29.7–49.1)	12.2(83)	36.1(26.5–47.1)	11.2(76)	39.5(29.1–50.9)	4.7(32)	40.6(25.2–58.2)						

Bold text and * indicates no overlap of 95%CIs between item and the ‘never’ category.

**Table 3:** Descriptive associations between forms of economic IPV and depressive symptoms and past four-week suicidal ideation.

		In the past 12 months how often did your partner stop you from getting a job, going to work, trading or earning money?		In the past 12 months how often did your partner take your earnings against your will?		In the past 12 months how often did your partner throw you out of the house?		In the past 12 months how often did your partner spend money on alcohol, tobacco or other things for himself when he knew you did not have enough for essential household expenses?		Any economic IPV in the past 12m		2 or more experiences of economic IPV in past 12m		Range of different forms of economic IPV (None or one versus two or more)	
		Exposure prevalence %(n)	Symptom mean /%	Exposure prevalence %(n)	Symptom mean /%	Exposure prevalence %(n)	Symptom mean /%	Exposure prevalence %(n)	Symptom mean /%	Exposure prevalence %(n)	Symptom mean /%	Exposure prevalence %(n)	Symptom mean /%	Exposure prevalence %(n)	Symptom mean /%
Depression	Past 12m never	77.8(529)	20.7(19.4–21.1)	90.2(613)	20.6(19.7–21.4)	81.6(555)	20.2(19.3–21.0)	61.6(419)	19.2(18.2–20.1)	47.8(325)	18.4(17.3–19.5)			44.7(516)	19.8(18.9–20.7)
	Past 12m once or more	22.2(151)	24.3(22.6–26.1)*	9.9(67)	26.8(24.1–29.5)*	18.4(125)	25.6(23.6–27.6)*	38.4(261)	24.4(23.1–25.7)*	52.2(355)	23.7(22.5–24.8)*			55.3(164)	25.6(23.9–27.2)*
	Past 12m never or once	87.7(596)	20.6(19.8–21.5)	96.5(656)	20.9(20.1–21.7)	90.0(612)	20.4(19.6–21.3)	69.1(470)	19.3(18.4–20.2)			56.3(383)	18.6(17.6–19.5)		
	Past 12m twice or more	12.4(84)	24.9(24.1–27.5)*	3.5(24)	28.5(23.4–33.6)*	10.0(68)	27.9(25.3–30.6)*	30.9(210)	25.4(23.9–26.9)*			43.7(297)	24.5(23.3–25.8)*		
	Frequency—never	77.8(529)	20.3(19.4–21.1)	90.2(613)	20.6(19.7–21.4)	81.6(555)	20.2(19.3–21.0)	61.6(419)	19.2(18.2–20.1)						
	Frequency—once	9.9(67)	23.6(21.1–26.1)	6.3(43)	25.7(22.8–29.0)*	8.4(57)	22.8(19.9–25.6)	7.5(51)	20.3(17.7–22.9)						
	Frequency—few	7.5(51)	22.6(19.7–25.6)	2.7(18)	26.4(21.1–31.7)*	4.6(31)	25.2(21.5–28.9)*	13.1(89)	23.3(21.0–25.5)*						
	Frequency—many	4.9(33)	28.5(24.3–32.7)*	0.9(6)	34.7(23.2–46.1)*	5.4(37)	30.2(26.7–33.8)*	17.8(121)	27.0(25.1-28-9)*						
Suicidal ideation	Past 12m never	77.8(529)	28.0(24.3–32.0)	90.2(613)	28.2(24.8–32.0)	81.6(555)	27.9(24.3–31.9)	61.6(419)	28.2(24.8–32.0)	47.8(325)	25.2(20.8–30.3)			44.7(516)	26.7(22.7–30.4)
	Past 12m once or more	22.2(151)	37.1(29.7–45.2)	9.9(67)	46.3(34.7–58.3)*	18.4(125)	39.2(31.0–48.1)	38.4(261)	46.3(34.7–58.3)*	52.2(355)	34.4(29.6–39.5)			55.3(164)	41.5(34.2–49.2)*
	Past 12m never or once	87.7(596)	28.7(25.2–32.5)	96.5(656)	29.0(25.6–32.6)	90.0(612)	28.6(25.1–32.4)	69.1(470)	29.0(25.6–32.6)			56.3(383)	25.6(21.4–30.3)		
	Past 12m twice or more	12.4(84)	39.3(29.4–50.1)	3.5(24)	58.3(38.4–75.9)*	10.0(68)	42.7(31.4–54.7)	30.9(210)	58.3(38.4–75.9)*			43.7(297)	35.7(20.5–41.3)		
	Frequency—never	77.8(529)	28.0(24.3–32.0)	90.2(613)	28.2(24.8–32.0)	81.6(555)	27.9(24.3–31.9)	61.6(419)	28.2(24.8–32.0)						
	Frequency—once	9.9(67)	34.3(23.9–46.5)	6.3(43)	39.5(26.1–54.8)	8.4(57)	35.1(23.9–48.3)	7.5(51)	39.5(37.9–54.8)*						
	Frequency—few	7.5(51)	29.4(18.6–43.3)	2.7(18)	61.1(37.9–80.2)*	4.6(31)	45.3(28.7–62.8)	13.1(89)	61.1(37.9–80.2)*						
	Frequency—many	4.9(33)	54.6(37.7–70.5)*	0.9(6)	50.0(16.8–83.3)	5.4(37)	40.5(26.09–6.8)	17.8(121)	50.0(16.8–83.3)						

Bolded text and *indicates no overlap between items 95%CI and the ‘never’ exposure category.

The most frequently reported form of economic IPV was “your partner spent money on alcohol, tobacco or other things for himself when he knew you did not have enough for essential household expenses” (38.4%) and the least frequent was taking a woman’s earnings against her will (9.9%) (Table 3). Approximately one fifth of all women reported being prevented from earning money (22.2%) or being thrown out of the house (18.4%). In total, 52.2% of the women reported any economic IPV in the past year, with 24.5% of the overall sample reporting two of more incidents, while 25.5% responded affirmatively to 2 or more of the 4 economic IPV items.

All measures of emotional and economic IPV we examined showed a consistent positive association with mean CESD-D scores (Tables 2 and 3). For each individual item used in the emotional IPV and economic IPV measures, the any vs none, and two or more vs none or one comparisons were significantly associated with increased CES-D scores, as indicated by the lack of overlaps between 95% CIs. A more detailed item-level examination of frequency using the four-level categories measured (never, once, few, many) showed little difference between the never and once categories. For all emotional IPV items, CES-D scores were higher for the many categories versus never, although there was overlap with 95% CI, but the difference between never and one experience, and never and few experiences, were not significant, apart from for women reporting ‘few’ experiences of their boyfriend hurting people they care about as a way of hurting them, where there was significant difference with women who reported no experience. For a few items, the pattern was slightly more complicated, without a clear ‘dose response’. For instance for suicidal ideation, for ‘being scared or intimidated’ women reporting this as ‘many’ had a lower prevalence of suicidal ideation than those reporting ‘few’ instances. There were also these issues for the items ‘being threatened with being hurt’ and ‘hurting others’.

A similar pattern was observed for economic IPV, whereby for all individual items any vs none, and two or more vs none or one comparisons were significantly different with no overlaps of 95% CI. In addition, women reporting many experiences of any individual item had significantly higher depressive symptoms compared to none, while the few category was marginal or non-significant.

All measures examined for each of emotional IPV, and economic IPV ‘full constructrs’ (any vs none, two or more events vs one or none) were significantly associated with increased CES-D scores.

The overall pattern of associations between emotional IPV, economic IPV, and self-reported suicidal ideation in the last four weeks were similar to those for CES-D scores, but differences were less often significant (Tables 2 and 3). The only individual item in the emotional IPV measure to show significant differences was “hurt people you care about as a way of hurting you, or damaged things of importance to you?” where there was no overlap between no experience and those reporting “few” experiences. None of the three composite measures of emotional IPV were significantly associated with suicidal ideation. For economic IPV, the individual items for “take your earnings against your will” and “spend money on alcohol, tobacco or other things for himself” were both associated with suicidal ideation across all summary and detailed measures, but the other two items were not significant. However, for all individual economic IPV items, there was not a clear ‘dose response’ of prevalence of suicidal ideation for never, once, few, many, although the combined measures showed this. The composite economic IPV measure for 2 or more types of economic IPV vs none or one, was significantly associated with suicidal ideation, while the any vs none measure was marginal. The composite variable for overall frequency of past year economic IPV was not associated with suicidal ideation.

Table 4 shows the combined four-level summary measures of emotional, economic, sexual, and physical IPV in a variety of permutations and their associations with mean CES-D scores. Summary measures examined, the highest mean CES-D score was observed for the category reporting women who experienced both emotional IPV or economic IPV, and physical IPV or sexual IPV, and in all cases this category was significantly different from the no IPV category.

**Table 4:** Descriptive associations between combined forms of IPV and depressive symptomology.

Row	Emotional IPV combined with physical, and sexual IPV	Exposure prevalence %(n)	Mean depressive symptoms (CI95%)	Economic IPV combined with physical, and sexual IPV	Exposure prevalence %(n)	Mean depressive symptoms (CI95%)	Emotional and/or economic IPV combined with physical, and sexual IPV	Exposure prevalence %(n)	Mean depressive symptoms (CI95%)
**1**	None	18.4(125)	18.5(16.7–20.3)	None	25.9(176)	17.3(15.8–18.8)	None	13.1(89)	17.5(15.4–19.6)
	Only emotional violence (1 or more)	22.1(150)	18.5(16.8–20.1)	Only economic violence (1 or more)	14.6(99)	20.5(18.6–22.4)	Only emotional and/or economic violence (1 or more)	27.4(186)	18.9(17.4–20.4)
	Emotional (1 or more) and physical IPV (1 or more)	56.0(38)	23.5(22.4–24.6)*	Economic (1 or more) and physical IPV (1 or more)	37.7(256)	24.9(23.6–26.3)*	Emotional and/or economic (1 or more) and physical (1 or more)	57.8(393)	23.3(22.2–24.3)*
	Physical only (1 or more)	3.5(24)	15.2(11.9–18.5)	Physical only (1 or more)	21.9(149)	19.7(18.2–21.2)	Physical only (1 or more)	1.8(12)	14.8(11.3–18.2)
**2**	None	19.0(129)	17.8(16.1–19.5)	None	39.7(270)	18.0(16.8–19.2)	None	13.5(92)	17.1(15.1–19.1)
	Only emotional violence (1 or more)	51.6(351)	20.0(18.9–21.0)	Economic only (1 or more)	30.9(210)	21.2(19.8–22.6)*	Emotional and/or economic only (1 or more)	57.1(388)	19.9(18.9–21.0)
	Emotional (1 or more) and sexual (1 or more)	26.5(180)	26.2(24.6–27.8)*	Economic (1 or more) and sexual (1 or more)	21.3(145)	27.3(25.5–29.1)*	Emotional and/or economic (1 or more) and sexual (1 or more)	28.1(191)	25.8(24.2–27.3)*
	Sexual only (1 or more)	2.9(20)	18.8(14.5–23.1)	Sexual only (1 or more)	8.1(55)	20.6(18.2–22.9)	Sexual only (1 or more)	1.3(9)	18.1(11.5–24.8)
**3**	None	16.3(111)	18.3(16.4–20.2)	None	23.5(160)	17.2(15.6–18.8)	None	12.1(82)	17.4(15.3–19.6)
	Emotional only (1 or more)	18.5(126)	18.0(16.1–19.9)	Economic only (1 or more)	11.3(77)	20.1(17.8–22.4)	Emotional and/or economic only (1 or more)	22.8(155)	18.5(16.9–20.2)
	Emotional (1 or more) and p/s (1 or more)	59.6(405)	23.3(22.3–24.4)*	Economic (1 or more) and p/s (1 or more)	40.9(278)	24.7(23.4–26.0)*	Emotional and/or economic (1 or more) and p/s (1 or more)	62.4(424)	23.1(22.1–24.1)*
	Physical and/or sexual only (1 or more)	5.6(38)	16.8(13.8–19.8)	Physical and/or sexual only (1 or more)	24.3(165)	19.6(18.1–21.0)	Physical and/or sexual only (1 or more)	2.8(19)	16.2(12.4–20.0)
**4**	None	28.8(196)	18.1(16.7–19.5)	None	36.8(250)	17.8(16.6–19.0)	None	20.7(141)	17.0(15.4–18.6)
	Emotional (2 or more)	22.7(154)	19.8(18.2–21.3)	Economic (2 or more)	19.6(133)	19.9(18.2–21.6)	Emotional and/or economic (2 or more)	30.7(209)	20.1(18.7–21.4)*
	Emotional (2 or more) and physical (2 or more)	43.8(298)	24.3(23.0–25.6)*	Economic (2 or more) and physical (2 or more)	29.0(197)	26.2(24.6–27.8)*	Emotional and/or economic (2 or more) and physical (2 or more)	45.6(310)	24.2(22.9–25.4)*
	Physical only (2 or more)	4.7(32)	17.6(14.7–20.6)	Physical only (2 or more)	14.7(100)	21.3(19.4–23.2)*	Physical only (2 or more)	2.9(20)	15.6(13.3–17.9)
**5**	None	30.7(209)	18.0(16.7–19.3)	None	50.6(344)	18.3(17.2–19.3)	None	22.4(152)	17.0(15.5–18.5)
	Emotional (2 or more)	48.1(327)	20.7(19.5–21.8)*	Economic (2 or more)	5.7(39)	21.2(18.1–24.3)	Emotional and/or economic (2 or more)	56.5(384)	20.7(19.6–21.7)*
	Emotional (2 or more) and sexual (2 or more)	18.4(125)	28.2(26.2–30.2)*	Economic (2 or more) and sexual (2 or more)	15.4(105)	29.0(26.9–31.1)*	Emotional and/or economic (2 or more) and sexual (2 or more)	19.9(135)	27.8(25.9–29.7)*
	Sexual only (2 or more)	2.8(19)	18.3(14.3–22.3)	Sexual only (2 or more)	28.2(192)	22.1(20.6–23.6)*	Sexual only (2 or more)	1.3(9)	13.8(9.3–18.3)
**6**	None	26.0(177)	18.0(16.6–19.5)	None	32.9(224)	17.7(16.4–19.0)	None	19.3(131)	17.1(15.5–18.8)
	Emotional (2 or more)	19.3(131)	19.5(17.7–21.2)	Economic (2 or more)	23.4(159)	19.7(18.2–21.2)	Emotional and/or economic (2 or more)	26.0(177)	19.8(18.2–21.3)
	Emotional (2 or more) and p/s (2 or more)	47.2(321)	24.1(22.9–25.3)*	Economic (2 or more) and p/s (2 or more)	31.3(213)	25.9(24.4–27.4)*	Emotional and/or economic (2 or more) and p/s (2 or more)	50.3(342)	23.9(22.8–25.1)*
	Physical and/or sexual only (2 or more)	7.5(51)	18.1(15.6–20.5)	Physical and/or sexual only (2 or more)	12.4(84)	21.0(18.9–23.2)	Physical and/or sexual only (2 or more)	4.4(30)	15.6(13.2–18.0)

Bolded and *Indicates no overlap between category’s 95%CI and the ‘none’ exposure.

Interesting differences emerge in looking at the emotional IPV or economic IPV only categories vs the physical and/or sexual IPV only categories. For 15 out of the 18 categorical variables tested, the category of emotional IPV or economic IPV only was associated with higher mean CES-D scores than the category of physical and/or sexual IPV only, the only exception being cases B4-6, looking at multiple events of economic IPV vs multiple events of physical and/or sexual IPV. Cases B4 and B5 also represent the only combinations where physical or sexual violence was significantly different from the no IPV referent, but the variable definition for these cases meant the women who had experience emotional IPV (but not economic) were coded in this group, and so these cases ultimately affirm the importance of emotional IPV as a correlate of CES-D score.

This is further affirmed by looking at cases C4-6, which show that the combined category for emotional and/or economic IPV only, associated with significantly higher mean CES-D scores than the categories for physical and/or sexual violence only. Further evidence for the importance of emotional IPV in mean CES-D score is found in case A5, where emotional IPV alone is associated with significantly higher CES-D scores than no IPV (noting that this category will include some women experiencing physical IPV without sexual IPV).

Table 5 reports associations between the 18 categorical variables and prevalence of past four week suicidal ideation. As with depressive symptoms, the highest prevalence of suicidal ideation in all combinations was where physical or sexual IPV was combined with emotional or economic IPV. However, only 6 out of 18 cases were significantly different from no IPV; all of these cases (B1-3, B5, C-45) involved economic IPV. As with depression above, suicidal ideation tended to be more common among women reporting only emotional and/or economical IPV compared to those reporting only physical and/or sexual IPV (15 out of 18 cases). Comparison of column B to column A affirms the suggestion that economic IPV may be particularly important for suicidal ideation, as cases involving economic IPV are generally associated with higher prevalence of suicidal ideation than emotional IPV. While the 95% CIs tend to overlap, the consistence and coherence of the difference across difference measurement categories is strongly suggestive.

**Table 5:** Descriptive associations between suicidal ideation and combinations of IPV.

Row	Emotional IPV combined with physical, and sexual IPV	Prevalence of exposure %(n)	% suicidal ideation (CI95%)	Economic IPV combined with physical, and sexual IPV	Prevalence of exposure %(n)	% suicidal ideation (CI95%)	Emotional and/or economic IPV combined with physical, and sexual IPV	Prevalence of exposure %(n)	% suicidal ideation (CI95%)
**1**	None	18.4(125)	27.2(20.1–35.7)	None	25.9(176)	22.7(17.1–29.5)	None	13.1(89)	23.6(15.9–33.6)
	Only emotional violence (1 or more)	22.1(150)	24.7(18.4–32.3)	Only economic violence (1 or more)	14.6(99)	31.3(22.9–41.2)	Only emotional and/or economic violence (1 or more)	27.4(186)	26.9(20.9–33.7)
	Emotional (1 or more) and physical IPV (1 or more)	56.0(38)	34.1(29.5–39.1)	Economic (1 or more) and physical IPV (1 or more)	37.7(256)	35.6(30.0–41.6)*	Emotional and/or economic (1 or more) and physical (1 or more)	57.8(393)	33.8(29.3–38.7)
	Physical only (1 or more)	3.5(24)	12.5(4.1–32.5)	Physical only (1 or more)	21.9(149)	28.2(21.5–36.0)	Physical only (1 or more)	1.8(12)	0.00(0.00–0.00)
**2**	None	19.0(129)	23.3(16.8–31.3)	None	39.7(270)	25.6(20.7–31.2)	None	13.5(92)	20.7(13.6–33.9)
	Only emotional violence (1 or more)	51.6(351)	29.1(24.5–34.1)	Economic only (1 or more)	30.9(210)	30.0(24.2–36.5)	Emotional and/or economic only (1 or more)	57.1(388)	29.1(24.8–33.9)
	Emotional (1 or more) and sexual (1 or more)	26.5(180)	36.1(29.4–43.5)	Economic (1 or more) and sexual (1 or more)	21.3(145)	40.7(33.0–48.9)*	Emotional and/or economic (1 or more) and sexual (1 or more)	28.1(191)	36.7(30.1–43.8)
	Sexual only (1 or more)	2.9(20)	35.0(26.6–33.6)	Sexual only (1 or more)	8.1(55)	23.6(14.2–36.7)	Sexual only (1 or more)	1.3(9)	22.2(5.5–58.2)
**3**	None	16.3(111)	25.2(18.0–34.1)	None	23.5(160)	22.5(16.7–29.6)	None	12.1(82)	23.2(15.3–33.6)
	Emotional only (1 or more)	18.5(126)	25.4(18.5–33.8)	Economic only (1 or more)	11.3(77)	31.2(21.8–42.4)	Emotional and/or economic only (1 or more)	22.8(155)	26.5(20.1–34.0)
	Emotional (1 or more) and p/s (1 or more)	59.6(405)	33.3(28.9–38.1)	Economic (1 or more) and p/s (1 or more)	40.9(278)	35.3(29.9–41.0)*	Emotional and/or economic (1 or more) and p/s (1 or more)	62.4(424)	33.5(29.1–38.2)
	Physical and/or sexual only (1 or more)	5.6(38)	23.7(12.8–39.7)	Physical and/or sexual only (1 or more)	24.3(165)	27.9(21.5–35.3)	Physical and/or sexual only (1 or more)	2.8(19)	10.5(2.6–33.9)
**4**	None	28.8(196)	29.1(23.1–35.8)	None	36.8(250)	25.6(20.6–31.4)	None	20.7(141)	24.8(18.4–32.7)
	Emotional (2 or more)	22.7(154)	26.0(19.6–33.5)	Economic (2 or more)	19.6(133)	25.6(18.9–33.7)	Emotional and/or economic (2 or more)	30.7(209)	29.7(23.9–36.2)
	Emotional (2 or more) and physical (2 or more)	43.8(298)	33.9(28.7–39.5)	Economic (2 or more) and physical (2 or more)	29.0(197)	37.1(30.6–44.0)	Emotional and/or economic (2 or more) and physical (2 or more)	45.6(310)	33.9(28.8–39.4)
	Physical only (2 or more)	4.7(32)	18.8(8.6–36.0)	Physical only (2 or more)	14.7(100)	33.0(24.5–42.7)	Physical only (2 or more)	2.9(20)	10.0(2.5–32.5)
**5**	None	30.7(209)	28.2(22.5–34.8)	None	50.6(344)	25.6(21.2–30.5)	None	22.4(152)	23.7(17.5–31.2)
	Emotional (2 or more)	48.1(327)	27.8(23.2–33.0)	Economic (2 or more)	5.7(39)	25.6(14.4–41.5)	Emotional and/or economic (2 or more)	56.5(384)	29.7(25.3–34.5)
	Emotional (2 or more) and sexual (2 or more)	18.4(125)	40.0(31.8–48.9)	Economic (2 or more) and sexual (2 or more)	15.4(105)	41.9(32.8–51.6)*	Emotional and/or economic (2 or more) and sexual (2 or more)	19.9(135)	39.3(31.4–47.8)*
	Sexual only (2 or more)	2.8(19)	21.1(8.1–44.7)	Sexual only (2 or more)	28.2(192)	32.3(26.1–39.2)	Sexual only (2 or more)	1.3(9)	11.1(1.5–50.6)
**6**	None	26.0(177)	25.2(18.5–33.4)	None	32.9(224)	26.8(21.4–33.0)	None	19.3(131)	25.2(18.5–33.4)
	Emotional (2 or more)	19.3(131)	32.2(25.7–39.4)	Economic (2 or more)	23.4(159)	23.9(17.9–31.2)	Emotional and/or economic (2 or more)	26.0(177)	32.2(25.7–39.4)
	Emotional (2 or more) and p/s (2 or more)	47.2(321)	32.2(27.4–37.3)	Economic (2 or more) and p/s (2 or more)	31.3(213)	35.7(29.5–42.3)	Emotional and/or economic (2 or more) and p/s (2 or more)	50.3(342)	32.2(27.4–37.3)
	Physical and/or sexual only (2 or more)	7.5(51)	13.3(5.1–30.8)	Physical and/or sexual only (2 or more)	12.4(84)	35.7(26.2–46.5)	Physical and/or sexual only (2 or more)	4.4(30)	13.3(5.1–30.8)

Bolded and *indicates no overlap between 95%CIs between exposure and none.

## Discussion

Our analysis clearly highlights the importance of emotional IPV and economic IPV in driving significant mental health impacts for women, and that these extend beyond those found with the current, narrower focus on physical and/or sexual IPV alone. While there is a small, but growing body of research on the health impacts of emotional IPV, and its differential impacts compared to physical and/or sexual IPV [2, 12, 26], there has not been a similar focus on economic IPV and its impact on women’s mental health and wellbeing.

The analysis supports the hypothesis that emotional IPV and economic IPV, have distinct health impacts in themselves. For both economic IPV and emotional IPV the frequency of exposure (Tables 2 and 3) increased the severity of health impact in terms of depressive symptoms and suicidal ideation. The breadth of exposure for economic IPV, and emotional IPV, was also associated with increased depressive symptoms. This reinforces research that has emphasized that the severity of IPV women experience is critical to understanding health impacts [30], however that research focused on physical IPV and sexual IPV. In terms of categorizing severe emotional IPV, or severe economic IPV, two or more exposures appears to be important for mental health impacts.

There were also differences between the mental health impacts of emotional IPV and economic IPV, despite them being strongly inter-related concepts, they did function as distinctive constructs and should be considered as such. While both emotional IPV and economic IPV had similar patterns around associations with depressive symptomology, there were stronger and more consistent associations between economic IPV and suicidal ideation (Tables 2–5), compared to emotional IPV. There remains very limited research on the health impacts of emotional IPV and economic IPV, and the lack of research on this is potentially missing a significant burden on women’s poor mental health.

This analysis suggests that emotional IPV and economic IPV should not be regarded as ‘lighter’ less ‘important’ forms of IPV when compared to physical and/or sexual IPV, in terms of mental health outcomes. Women in this study who experienced only emotional, or economic IPV reported more depressive symptoms and were more likely to report suicidal ideation than those who experienced physical or sexual IPV only. Major depressive disorders (MDD) for women are common globally, with an estimate past year prevalence of 5.5%, and are associated with increasing death related to physical injuries, self-harm and suicide [31]. Moreover, MDD has multiple negative health outcomes, including increased non-adherence to medical treatment [32], increased risk of physical poor health [33, 34], and HIV-acquisition [35].This clearly suggests that given they independently drive women’s poor mental health, working to reduce these in prevention and intervention efforts needs to be expanded.

Finally, the analysis showed that the highest levels of depressive symptoms and prevalence of suicidal ideation, were seen amongst women who experienced emotional IPV or economic IPV, combined with physical IPV or sexual IPV (Tables 4 and 5), supporting a limited body of evidence showing similar outcomes [26]. The combined forms of IPV, with significant health impacts, are likely to be indicative of severity of IPV, that is severe physical or sexual IPV is unlikely to occur outside of emotional IPV and/or economic IPV.

There are several important implications of the analysis presented here for policy, and for research and evaluation. First, we do not need to measure all forms of emotional IPV and economic IPV to see a mental health impact. The addition of multiple items to questionnaires has often been considered as overly burdensome for studies, and not capturing any distinct impact on women’s health, beyond what has already been captured through assessing physical and sexual IPV. Given the very high prevalence of any emotional IPV and economic IPV and overlaps of types, the returns on measuring additional forms will not yield high returns when viewed in terms of having many more women being ‘exposed’. As has been shown, emotional IPV and economic IPV have distinct mental health impacts over and above physical and sexual IPV. As such their inclusion is important, but the analysis shows that a relatively small set of items can lead to capturing of these health impacts, particularly when combined with other forms of IPV.

Second, we do not need large numbers of indicators in scales to capture health impacts. As shown, measures of ever/never exposures, and in particularly two or more exposures (compared to none or one) capture the majority of mental health impact for women for emotional IPV or economic IPV. As such, it should be possible to derive a smaller sub-set of items that capture the majority of burden that women experience in terms of emotional and economic IPV.

Finally, given that emotional IPV and economic IPV have distinctive outcomes in terms of mental health, as well as being clear indicators of severity of impact, when combined with physical and sexual IPV, it is critical that these are included as trial outcomes in interventions seeking to prevent IPV. At a practical level reducing all forms of IPV that have an impact on mental health is crucial, and therefore as emotional IPV and economic IPV have a distinct and separate impact to physical and/or sexual IPV, the impact of interventions on these should be assessed. In addition, other studies have hinted that interventions may have different impacts on different forms of IPV. For instance, an evaluation of Oportunidades in Mexico showed a reduction in physical IPV amongst female recipients, but increases in emotional IPV and increases in threats of violence, however these were not statistically significant [36].

This paper has a number of limitations. First, data is cross-sectional and as such temporality of relationships cannot be ascertained, and there is evidence that the relationship between IPV and depression is bidirectional [6, 37]. Second, depression and suicidal ideation were assessed using screening tools, rather than through clinical assessments. There was also a very high prevalence of all forms of IPV, which may have shaped findings, but it is not clear that this would cause bias. We also did not exhaustively develop economic or emotional IPV measures for this population, which could have implications for the analysis. Specifically, many of the women did not live with their partners, and many of the economic IPV questions presuppose a level of economic dependence we did not screen for and an assumption of a nuclear household. However, in this population, anthropological research has emphasized romantic love and economic support are inter-connected [38, 39] and as such, it seems reasonable to assume a level of economic dependence. In addition, the sample size was not huge, and as such while there were clear graduations in increased health impacts, there was often overlap of 95% confidence intervals, and large confidence intervals, limiting interpretation of findings. In addition, because of the limitations of sample size, we were unable to create entirely ‘clean’ referent categories with women who experienced no emotional, economic, sexual or physical IPV, nor did we exclude women who reported no partners in the past year. Both of these would have pushed towards the null hypothesis, suggesting our results are relatively robust. As the sample size was determined by the primary trial analysis [22], this may limit the current analysis. Specifically, a number of outcomes assessed in this paper, such as suicidal ideation, while common, were relatively infrequent in some of the combinations shown, leading to small cell sizes, and large and overlapping 95% confidence intervals and a lack of precision in the analysis. It could be for this reason that not all individual items showed a clear dose response for the scales. Finally, study participants were recruited into an intervention trial, and it may be that this shaped the high prevalence of IPV reported and certainly the analysis cannot be generalized outside of this population. These limitations suggest a need for replication in population representative data, and in larger data sets.

## Conclusion

The role of emotional IPV and economic IPV in driving health impacts of women is rarely considered, although there is growing interest in emotional IPV, specifically around its consideration as an SDG indicator. The paper clearly highlights that the different forms of IPV (emotional, economic, physical, and sexual) while strongly overlapping also have distinctive health outcomes that need to be considered in research, intervention and policy as such. It is very important that this research is replicated in other datasets and a view on the main findings across multiple settings is reached as this can substantially advance knowledge and understanding of this important area of women’s experience of IPV.

## Supporting information

S1 Table Wording of items of emotional IPV and economic IPV from instruments used in three major population-based research programmes. (DOCX)

S1 File Baseline dataset for Stepping Stones and Creating Futures trial for this analysis. (CSV)
