# Supplementary material for: Emotional and economic intimate partner violence as key drivers of depression and suicidal ideation: A cross-sectional study among young women in informal settlements in South Africa
Source: PLoS One. 2018 Apr 16;13(4):e0194885. doi: 10.1371/journal.pone.0194885 (PMC5901771; doi:10.1371/journal.pone.0194885)
Supplement: S1 Table — (DOCX) [file pone.0194885.s001.docx]

| WHO MCS (2005,[[7](#_ENREF_7)]) | UNMCS (2010, [[8](#_ENREF_8)]) | DHS Domestic Violence Module (2017, [[18](#_ENREF_18)]) |
| --- | --- | --- |
| Emotional violence | | |
| I want you to tell me if your current husband/partner, or any other partner, has ever done the following things to you:  Insulted you or made you feel bad about yourself? | Has a current or previous husband or boyfriend ever insulted you or made you feel bad about yourself? | Did your (last) (husband/partner) ever: say or do something to humiliate you in front of others? |
| I want you to tell me if your current husband/partner, or any other partner, has ever done the following things to you:  Belittled or humiliated you in front of other people? | Has a current or previous husband or boyfriend ever belittled or humiliated you in front of other people? | Did your (last) (husband/partner) ever: threaten to hurt or harm you or someone you care about? |
| I want you to tell me if your current husband/partner, or any other partner, has ever done the following things to you:  Did things to scare or intimate you on purpose (e.g. by the way he looked at you, by yelling and smashing things)? | Has a current or previous husband or boyfriend ever done things to scare or intimidate you on purpose for example, by the way he looked at you, by yelling or smashing things? | Did your (last) (husband/partner) ever: insult you or make you feel bad about yourself? |
| I want you to tell me if your current husband/partner, or any other partner, has ever done the following things to you:  Threaten to hurt you or someone you care about? | Has a current or previous husband or boyfriend ever threatened to hurt you? |  |
|  | Has a current or previous husband or boyfriend ever hurt people you care about as a way of hurting you, or damaged things of importance to you? |  |
| Economic Violence | | |
| Are you able to spend the money you earn you earn how you want yourself, or do you have to give all or part of your money to your husband/partner | Has a current or previous husband or boyfriend ever prohibited you from getting a job, going to work, trading, earning money or participating in income generation projects? |  |
| Have you ever given up/refused a job for money because your husband/partner did not want you to work? | Has a current or previous husband or boyfriend ever taken your earnings from you? |  |
| Has your husband/partner ever taken your earnings or savings against your will? | Has a current or previous husband or boyfriend ever thrown you or your children out of the house where you were living? |  |
| Does your husband/partner ever refuse to give you money for household expenses, even when he has money for other things? | Has a current or previous husband or boyfriend ever refused to give you money you needed for household expenses even when he has money for other things? |  |
